# Diagnostic evidence cooperatives: bridging the valley of death in diagnostics development

**DOI:** 10.1186/s41512-018-0030-9

**Published:** 2018-06-18

**Authors:** Ann Van den Bruel, Gail Hayward

**Affiliations:** 1 NIHR Diagnostic Evidence Cooperative Oxford, Nuffield Department of Primary Care Health Sciences, University of Oxford, Radcliffe Primary Care Building, 32 Woodstock Road, Oxford, OX2 6GG UK; 2 Academic Centre for Primary Care, KU Leuven, Belgium

**Keywords:** Diagnosis, Primary care, Research and development

## Abstract

**Background:**

Diagnostic tests’ impact on patient outcomes and health processes is potentially large, and proper evaluations before widespread adoption are warranted. Such evaluations are challenged by the fact that tests can have multiple purposes, in different clinical pathways, with different roles.

**Body:**

The National Institute for Health Research (NIHR) established four Diagnostic Evidence Cooperatives (DEC) in 2013, across England. The aim of these DECs was to facilitate the development and evaluation of clinically relevant in vitro diagnostics, by offering methodological expertise and access to real-life settings for evaluations in patients. In this commentary, we discuss our experience over the past 4 years.

**Conclusion:**

The interaction of industry, researchers, and clinicians has proven to be very worthwhile.

## Background

Diagnostic tests are pivotal to health care and encompass a wide range of modalities from history taking or clinical examination to imaging, blood tests, and pathology. We use tests for a variety of reasons, such as risk prediction, early detection, diagnosis, monitoring, and prognosis. In contrast to treatments, the potential impact of tests is not always appreciated. Tests obviously can have indirect impact by changing treatment decisions, but they can also have direct impact including the psychological and emotional effects of diagnostic labelling (be it positive or negative) and complications of tests such as bowel perforation from colonoscopy or radiation exposure from imaging. Whether or not the test is worthwhile depends on the balance between benefits and harms, which means both need to be assessed and quantified. Several frameworks have been proposed that describe the different types of evidence that is needed for new diagnostic tests [1], and although there are some differences between these frameworks, most include evidence on technical accuracy (can it measure what it should measure in lab conditions), diagnostic accuracy (does it measure what it should measure in patients), clinical utility (does it improve patient outcome), and is it worth it (cost-effectiveness) [2–4].

## Body

As a result, evidence is required for tests to be allowed onto the market and recommended for routine care. In the past, regulatory approvals focused mainly on safety, with many being able to self-certify for CE marking which secures market access in the EU and beyond. In the new EU Regulation for In Vitro Diagnostics (Regulation 2017/746), published in May 2017 and coming into full effect in 2022, more evidence will be required to demonstrate safety and accuracy depending on the test’s risk level, which will pose additional challenges to in vitro diagnostic (IVD) developers. National bodies such as the National Institute for Health and Care Excellence (NICE) already have raised the bar for diagnostic tests to be recommended, by demanding evidence on cost-effectiveness which includes impact on patient outcome albeit this may be indirect from linking diagnostic accuracy evidence with pre-existing evidence on treatment efficacy. On average, it takes 9 years for evidence to accumulate on a test’s ability to diagnose a condition through to its efficacy and cost-effectiveness [5]. This contrasts with the increasingly rapid cycles of test development, with new tests offering greater precision, faster results, or reduced complexity, allowing use in community settings.

Many diagnostic tests have multiple purposes, a feature which complicates test evaluation. For example, HbA1c is used for both monitoring diabetic treatment response and initial diagnosis of diabetes. New regulations demand the intended role be clear, showing accuracy for that role in a relevant population because accuracy may change with changes in the spectrum of disease, an issue that is particularly relevant for primary care [6, 7]. Moreover, depending on whether the test will be used primarily to rule out a condition early on in the clinical pathway or to confirm a condition before starting treatment, and the consequences of missing cases or wrongfully labelling someone as diseased, different diagnostic properties will be required to minimize either false negatives or false positives respectively. Clarifying this early on by interacting with the clinical community could save test developers time and money and improve the chances of adoption at the end of the development pathway.

In routine care, every innovative test will be part of a clinical pathway, and its effect will largely depend on the nature of this clinical pathway and how effective this existing pathway is without additional tests. In the early phases of diagnostic test evaluation, describing this clinical pathway is of the utmost importance. It will clarify the goal of the new test and its associated desired diagnostic properties. It will also help to identify the minimum requirements for a cost-effective test. Clinical pathways are mostly established by expert opinion, clinical guidelines, and best practice. However, real-life pathways can deviate significantly from these ‘ideal’ pathways. Patients undergoing chemotherapy have been shown to follow a large number of pathways, almost equal to the number of patients: in effect, an individual pathway [8]. This indicates that a more rigorous approach is needed to clearly define clinical pathways, with variation between patients taken into account when assessing potential effects. Innovative tests that do not fit in the pathway may require service redesign which not only is more difficult to achieve but also makes it more difficult to predict the effects of the new test. Pilot studies of such service redesign will mitigate risks and provide the required evidence on uptake, accuracy, and impact. In effect, such disruptions may prove to be highly effective, as a newly established unit for frail elderly using point-of-care testing has shown [9].

For all these reasons, it should come as no surprise that the transition from a successful proof of concept to a commercially available test that is ready for implementation in routine care has been shown to be challenging, especially for test developers whose expertise understandably lies in technological development rather than clinical epidemiology. This ‘valley of death’ could be overcome by providing test developers with access to expertise in diagnostic test evaluation, information on clinical needs, and real-life settings for evaluations in patient populations. To this end, the UK Department of Health and the National Institute for Health Research (NIHR) funded four Diagnostic Evidence Cooperatives (DECs) in 2013, based in Newcastle, Leeds, Oxford, and London. Our Oxford DEC has focused on in vitro diagnostics for primary care, where currently diagnostic opportunities are relatively limited or require a referral to a laboratory, imaging facility, or secondary care. Our team consists of general practitioners and researchers with expertise in diagnostic test evaluation, health economists, statisticians, and qualitative researchers.

Over the past 4 years, we have met with 54 companies to advise them on the test’s potential role in primary health care, and the next steps in the evidence accumulation. We have been amazed at the technological possibilities under development, which hold great promise for the future. Our Horizon Scanning program, which has used a standardized published methodology [10] to produce 48 reports on innovative diagnostic technology relevant for primary care, suggests diagnosis in primary care could undergo very substantive changes over the next decade (https://www.community.healthcare.mic.nihr.ac.uk/about-us/horizon-scanning-1). However, a review of all of our Horizon Scans showed that for most tests evidence on impact is lacking [5]. Over the past 5 years, we also came across tests that offered only small improvements (such as a 5-min improvement in turnaround time), tests looking for a problem (tests for self-limiting infections that require no treatment), and tests that are technically possible but clinically probably not desirable because of the negative impact they may have (screening for an illness for which no treatment is yet available). Interactions were most useful when companies approached us at a stage where adaptations were possible, i.e. to adapt the new device to routine care practicalities but more importantly to the clinical need. Additionally, we have signposted companies to researchers working in other clinical settings where we felt the technology would be more useful, to funding agencies, and set up collaborations and joint grant applications to set up clinical studies and health economic modeling studies to estimate accuracy and impact.

Clinicians have generally shown appetite for new diagnostic tests. An international survey of general practitioners indicated a need for point-of-care tests for acute and potentially serious conditions such as myocardial infarction and pulmonary embolism to guide referrals, for infections to guide treatment in primary care, and for chronic conditions such as diabetes and anticoagulant use to monitor treatment [11]. Strikingly, point-of-care tests are already available for most of these conditions but uptake has been either non-existent or haphazard. There are several explanations for this including the fact that tests are not fit for purpose (they take too long, require sample handling that is beyond what can be achieved in primary care, the tests’ scope is too limited). In addition, general practices may not have clinical governance schemes, including legal, regulatory, and quality control issues, and experience financial barriers for implementation. GPs have also expressed concerns about accuracy of point-of-care tests, losing clinical skills from over-relying on tests, and tests’ limited usefulness in primary care settings [12]. Aligning clinical need with test development will require a myriad of information types, such as surveys to explore clinician preferences, data analyses to identify diagnostic bottlenecks, and qualitative studies with clinicians, patients, and other stakeholders to explore new opportunities.

In January 2018, all four DECs have reincarnated as some of the 11 different organizations, which have been designated NIHR Medtech and In Vitro Diagnostics Cooperatives. Our new incarnation, the NIHR Community Healthcare Medtech and IVD Cooperative, will learn from the past 4 years’ work by engaging with companies far earlier in the pipeline of development of diagnostic tests, in addition to the ongoing remit of supporting the generation of evidence for commercially available diagnostics. Working with companies who may not yet have chosen a target pathway for their diagnostic will allow us to support the development of tests which fill a genuine clinical need and to harness the potential of exciting new technologies to improve patient outcomes in community healthcare, with particular focus on chronic disease, infections, and diagnostics which facilitate ambulatory care. In addition, we have expanded our remit from purely IVDs to all diagnostic technologies, recognizing that often new pathways of care will include multiple tests of different modalities.

## Conclusions

The DECs have been a very worthwhile experiment in asking two very different types of people, industries, and researchers, to explore each other’s perspectives and to develop a shared approach to achieving the goals of introducing new relevant diagnostics to improve patient care. Our DEC has explored clinical needs in current routine practice, collated existing evidence on innovative diagnostic tests, highlighted evidence gaps in the current evidence trajectory, and facilitated evidence accumulation by providing access to expertise, patient populations, and clinical studies. The DECs, with their mandate to support industry in evidence generation, are unique in life sciences. Other fields of industrial development could well benefit from similar approaches.

## Abbreviations

DEC: Diagnostic evidence cooperative
IVD: In vitro diagnostic
NICE: National institute for care and health excellence
